# Stratification of Digestive Cancers with Different Pathological Features and Survival Outcomes by MicroRNA Expression

**DOI:** 10.1038/srep24466

**Published:** 2016-04-15

**Authors:** Senwei Tang, William K. K. Wu, Xiangchun Li, Sunny H. Wong, Nathalie Wong, Matthew T. V. Chan, Joseph J. Y. Sung, Jun Yu

**Affiliations:** 1 Institute of Digestive Disease and Department of Medicine & Therapeutics, State Key Laboratory of Digestive Disease, LKS Institute of Health Sciences, CUHK Shenzhen Research Institute, The Chinese University of Hong Kong, Hong Kong; 2 Department of Anaesthesia and Intensive Care, The Chinese University of Hong Kong, Hong Kong; 3 Department of Anatomical and Cellular Pathology, The Chinese University of Hong Kong, Hong Kong

## Abstract

MicroRNAs (miRNAs) are aberrantly expressed in virtually all cancer types, including digestive cancers. Herein, we aggregated and systematically analyzed miRNA expression profiles of 1765 tumor samples, including esophageal, gastric, liver, pancreatic, colon and rectal cancers, obtained through small RNA sequencing by The Cancer Genome Atlas. We found that digestive cancers of different tissue origins could be differentiated according to their miRNA expression profiles. In particular, esophageal squamous cell carcinoma and esophageal adenocarcinoma exhibited distinct miRNA expression patterns. Thirteen (e.g. miR-135b, miR-182) and sixteen (e.g. miR-139, miR-133a-1, miR-490) miRNAs were commonly upregulated and downregulated in more than four cancer types, respectively. Pertinent to pathological features, low miR-181d expression was associated with microsatellite instability in colon and gastric cancers whereas low miR-106a expression was associated with hepatitis B virus infection in hepatocellular carcinoma. Progression in colon cancer could also be predicted by low let-7f-2 and high miR-106a expression. Molecular subtypes with distinct prognostic outcomes independent of tumor-node-metastasis staging were identified in hepatocellular carcinoma and colon cancer. In total, 4 novel and 6 reported associations between specific miRNAs and patients’ survival were identified. Collectively, novel miRNA markers were identified to stratify digestive cancers with different pathological features and survival outcomes.

Digestive cancers, including esophageal, gastric, liver, pancreatic and colorectal cancers, are collectively a major cause of cancer morbidity and mortality in the world, posing a heavy burden on the healthcare system^1^. Understanding their molecular pathogenesis is key to improving risk prediction, prognostication and treatment^2^. The application of next-generation sequencing technologies, such as whole-genome and RNA sequencing, has enabled the depiction of mutational and transcriptomic landscapes of digestive cancers at unprecedentedly high resolution^3^,^4^,^5^. Nevertheless, challenges remain for the clinical utilization of these “big data” for molecular typing. To this end, stratifying patients with different disease outcomes using omics data is an area of active investigation^6^. For instance, Cristescu *et al.* used gene expression data to describe four molecular subtypes of gastric cancer that are linked to distinct patterns of molecular alterations, disease progression and prognosis^7^. Using exome sequencing and targeted capture sequencing, our group also identified a five-gene mutational signature, which predicts patients’ overall survival independent of tumor-node-metastasis (TNM) staging in colorectal cancer^8^. It is propitious that more in-depth analysis of existing omics data in association with clinicopathological information will give rise to novel molecular biomarkers that may translate into clinical benefits.

MicroRNAs (miRNAs) are a group of small non-coding RNAs that are ~22 nucleotides in length. miRNAs are aberrantly expressed in virtually all types of human cancers, including digestive cancers^9^,^10^,^11^,^12^, in which they could alter cellular phenotypes, such as proliferation, apoptosis and invasiveness, through their interactions with intracellular signaling networks and thereby functioning as proto-oncogenes or tumor suppressor genes. Importantly, some miRNAs have been shown to correlate with cancer progression and thus may be used as prognostic markers^13^. However, owing to the use of different profiling methods and the limited sample size, studies often yield inconsistent results regarding the functional roles or prognostic values of miRNAs^14^,^15^,^16^. In recent years, the identification of dysregulated miRNAs in human cancers has been facilitated by the application of next-generation sequencing^17^,^18^. Nevertheless, the use of the generated datasets for discovery of novel miRNA markers for clinical utilization, particularly prognostication, has not yet been achieved.

Here we report an integrative analysis of digestive cancers by their miRNA expression profiles obtained from The Cancer Genome Atlas (TCGA). We demonstrated that miRNA expression profiles could be used to differentiate digestive cancers of different tissue origins. Importantly, we identified molecular subtypes and specific miRNAs that were associated with clinicopathological features, including patients’ survival.

## Results

### miRNA dysregulation patterns in digestive cancers

**Figure 1:**
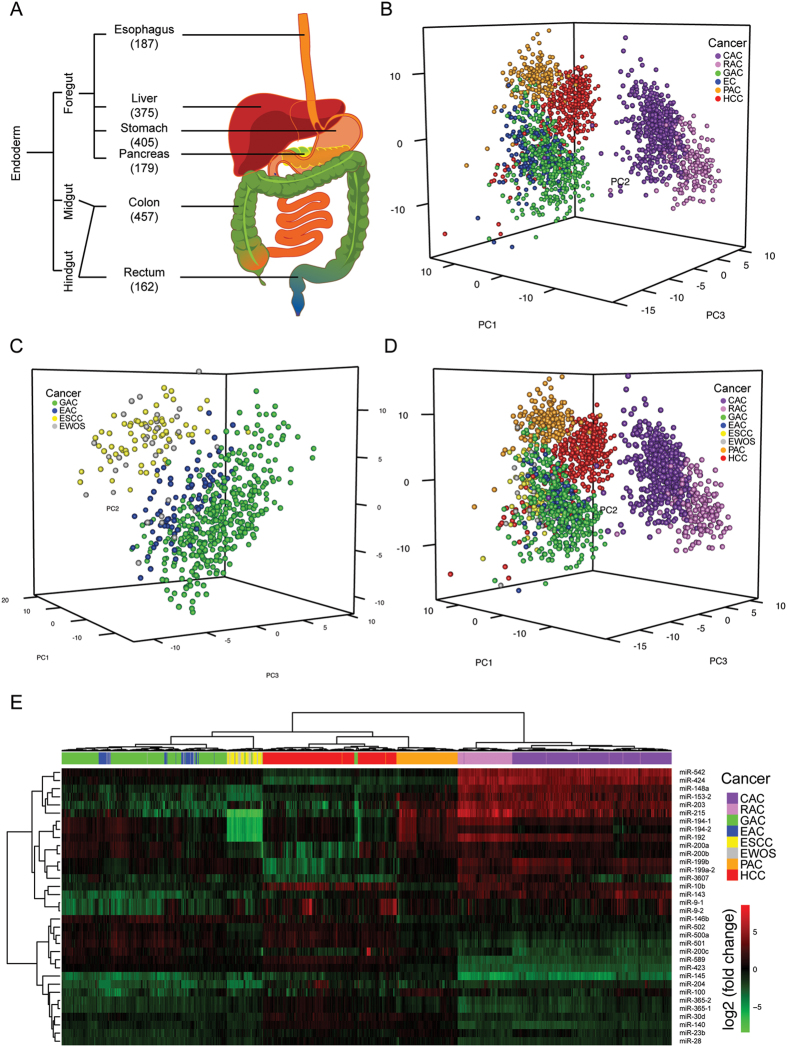
miRNA expression profiles of 1765 digestive cancer samples. (**A**) Small RNA sequencing data of 1765 digestive cancer samples were collected from TCGA. Embryonic origin and number of samples of each digestive cancer were shown. (**B–D**) Principal component analyses were performed for (**B**) six major digestive cancers, (**c**) esophageal and gastric cancers, and (**D**) seven reclassified digestive cancers. (**E**) Hierarchical clustering of all tumor samples was performed using expression profiles of 34 random forest-selected miRNAs. CAC, colon adenocarcinoma; RAC, rectal adenocarcinoma; GAC, gastric adenocarcinoma; EAC, esophageal adenocarcinoma; ESCC, esophageal squamous cell carcinoma; EWOS, esophageal cancer not otherwise specified; PAC, pancreatic adenocarcinoma; HCC, hepatocellular carcinoma.

**Figure 2:**
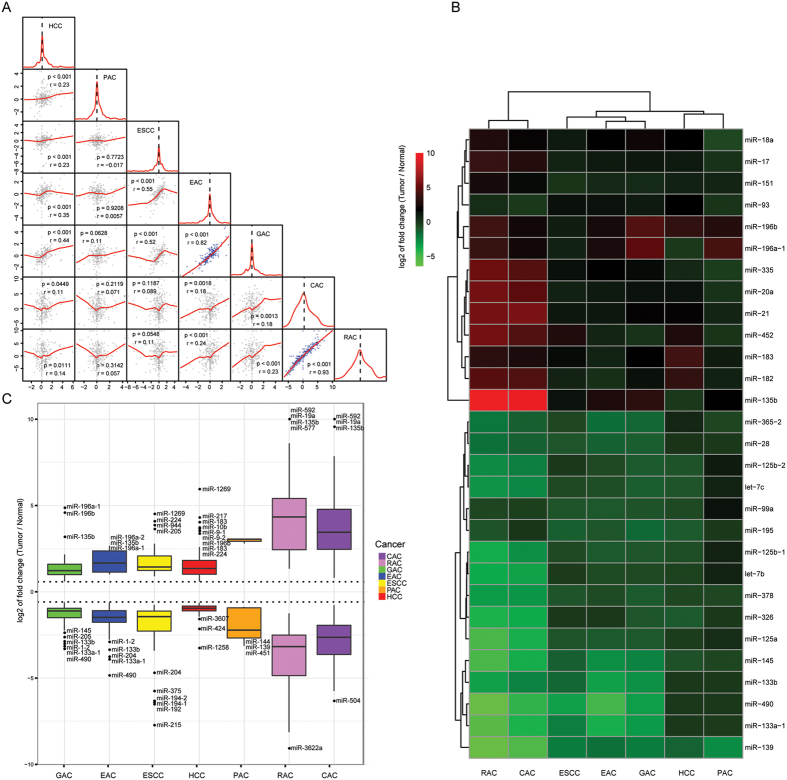
Similarities across seven digestive cancers. (**A**) Pairwise correlations between samples from seven digestive cancer types using miRNA differential expression profiles show the degree of similarities of miRNA dysregulation across cancers. Significant correlated pairs are indicated by blue dots. (**B**) Significantly differentially expressed miRNAs shared by more than four types of digestive cancer were subject to heatmap analysis. (**C**) Outlier miRNAs with extreme dysregulation in digestive cancers were shown. CAC, colon adenocarcinoma; RAC, rectal adenocarcinoma; GAC, gastric adenocarcinoma; EAC, esophageal adenocarcinoma; ESCC, esophageal squamous cell carcinoma; PAC, pancreatic adenocarcinoma; HCC, hepatocellular carcinoma.

PCA using the differential miRNA expression data of 1765 tumor samples from six major digestive cancers, namely esophageal cancer, hepatocellular carcinoma (HCC), gastric adenocarcinoma, pancreatic adenocarcinoma, colon adenocarcinoma and rectal roughly divided samples into 5 subgroups consistent with their tumor origins but esophageal cancer samples were mixed with gastric adenocarcinoma (Fig. 1A,B). Further clustering using PCA divided esophageal cancer and gastric adenocarcinoma samples into two subgroups, in which one subgroup was dominated by esophageal squamous cell carcinoma (ESCC) while the other consisted of esophageal adenocarcinoma (EAC) and gastric adenocarcinoma (Fig. 1C), thereby yielding a total of seven distinct clusters in PCA (Fig. 1D). Unsupervised hierarchical clustering showed a pattern in accord with that of PCA (Fig. 1E; Supplementary Figure 2 and 3). Pairwise correlations showed a high concordance between EAC and gastric adenocarcinoma (r > 0.86; *p* < 0.001) as well as colon and rectal adenocarcinomas (r = 0.95; *p* < 0.001) (Fig. 2A). Thirteen upregulated miRNAs (e.g. miR-135b, miR-182) and 16 downregulated (e.g. miR-139, miR-133a-1, miR-490) showed dysregulation of >1.5-fold in the same direction in more than four types of digestive cancer (Fig. 2B). Common outlier miRNAs with extreme dysregulation in seven types of digestive cancer were also identified (Fig. 2C).

### miRNAs associated with MSI and HBV

**Figure 3:**
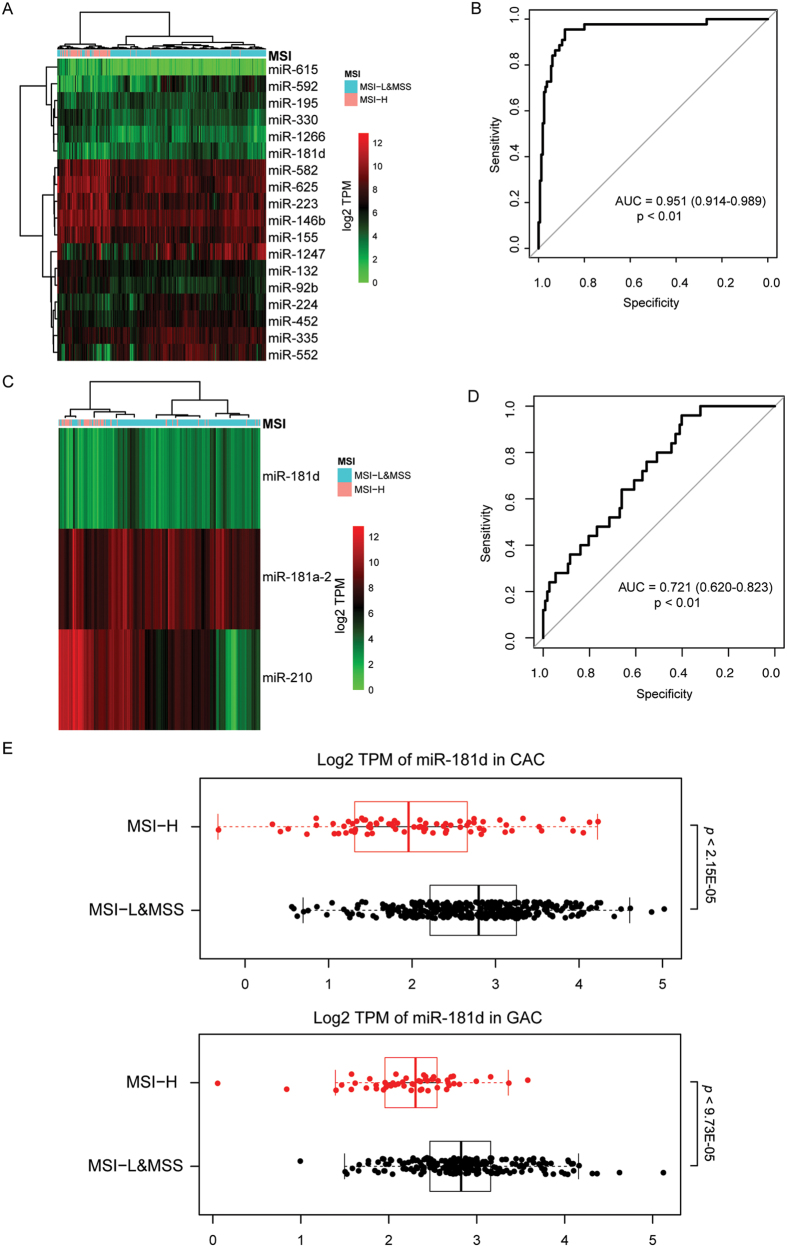
Correlation between miRNA expression and microsatellite instability (MSI) status. (**A–D**) Random forest and receiver-operating characteristic (ROC) curve were used to select miRNAs with greatest contribution to MSI classification and for performance evaluation in (**A**,**B**) in colon adenocarcinoma and (**C**,**D**) gastric adenocarcinoma. (**E**) miR-181d expression was significantly lowered in MSI-H samples than non-MSI-H samples in colon and gastric adenocarcinomas. AUC, area under the ROC curve. CAC, colon adenocarcinoma; GAC, gastric adenocarcinoma; MSI-H, MSI-high; MSI-L, MSI-low; MSS, microsatellite stable.

The correlation between miRNA expression and pathological features was examined. One notable finding was that miRNA expression profiles of colon adenocarcinoma with high microsatellite instability (MSI-H) were visibly different from those of low MSI (MSI-L) and microsatellite-stable (MSS) samples as shown in hierarchical clustering (Fig. 3A; Supplementary Figure 3). ROC curve was used to evaluate the performance of random forest-selected miRNAs in classifying MSI-H from non-MSI-H samples, which yielded an AUC of 0.951 (Fig. 3B; 95% CI: 0.914–0.989; *p* < 0.01). Hierarchical clustering of differentially expressed miRNAs obtained a similar result in gastric adenocarcinoma (Fig. 3C,D; AUC: 0.721; 95% CI: 0.620–0.823; *p* < 0.01). In both gastric and colon adenocarcinomas, miR-181d expression levels were significantly lower in MSI-H samples than in non-MSI-H samples (Fig. 3E). Another interesting finding was the association between hepatitis B virus (HBV) infection and miRNA expression profiles in HCC. As shown in Supplementary Figure 4A, PCA could marginally separate HBV form non-HBV samples. In particular, miR-106a was found to have the greatest contribution to HBV status classification (Supplementary Figure 4B) and its expression was significantly higher in the non-HBV group than in the HBV group (Supplementary Figure 4C; *p* < 0.001; FDR < 0.01).

### miRNAs associated with disease progression in colon adenocarcinoma

**Figure 4:**
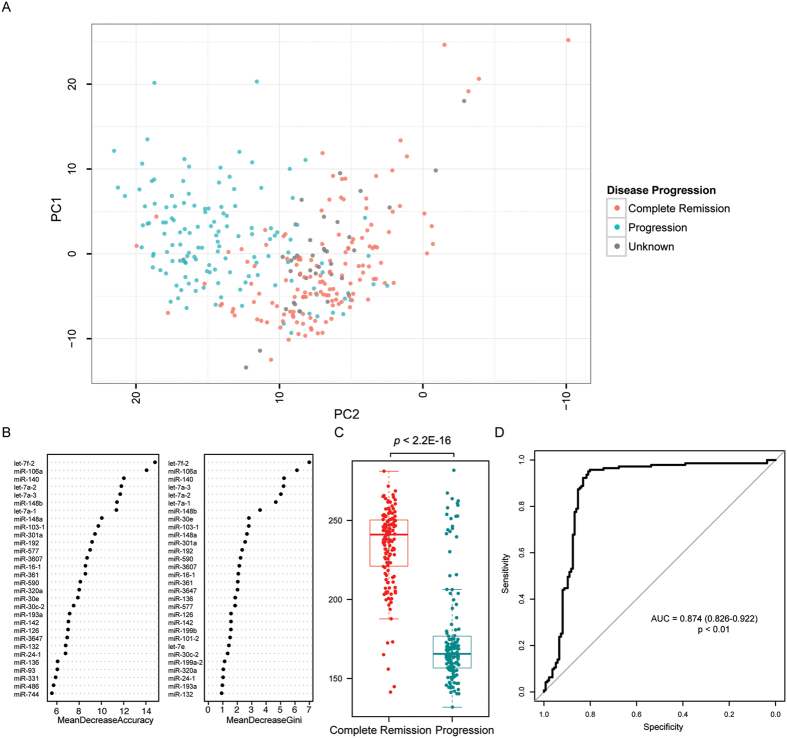
miRNAs associated with disease progression in colon adenocarcinoma (**A**) Principal component analysis identified miRNA expression pattern in colon adenocarcinomas with disease progression was different from those with complete remission. (**B**) Random forest was used to select miRNAs with greatest contribution to disease status classification. (**C**) A formula was constructed to calculate the ∆s score to predict the likelihood of disease progression. (**D**) Receiver-operating characteristic curve was used to evaluate the performance of the constructed formula.

To identify prognostic markers, the correlation between miRNA expression and clinical outcomes was assessed. In this respect, we observed that miRNA expression profiles of colon adenocarcinoma with complete remission were remarkably different from those with disease progression (Fig. 4A). Of note, treated samples (e.g. by chemotherapy or radiotherapy) were omitted in this analysis to eliminate the potential interference. Random forest algorithm was applied to pinpoint miRNAs with the greatest contribution to the disease status (Fig. 4B). In this regard, let-7f-2 and miR-106a could clearly separate complete remission samples from progression samples (Supplementary Figure 5). On the basis of this analysis, we developed a miRNA-based formula to predict disease progression in untreated colon adenocarcinoma: ∆s = 22.22 *log_2_(TPM of let-7f-2) + (−13.93)*log_2_(TPM of miR-106a). As shown in Fig. 4C,D, the smaller the ∆s was, the higher chance the patient had disease progression (AUC = 0.874; *p* < 0.01). At a cutoff of 207.9, ∆s could predict disease progression with a sensitivity of 0.839 and a specificity of 0.853.

### miRNA patterns predictive of survival in HCC and colon adenocarcinoma

**Figure 5:**
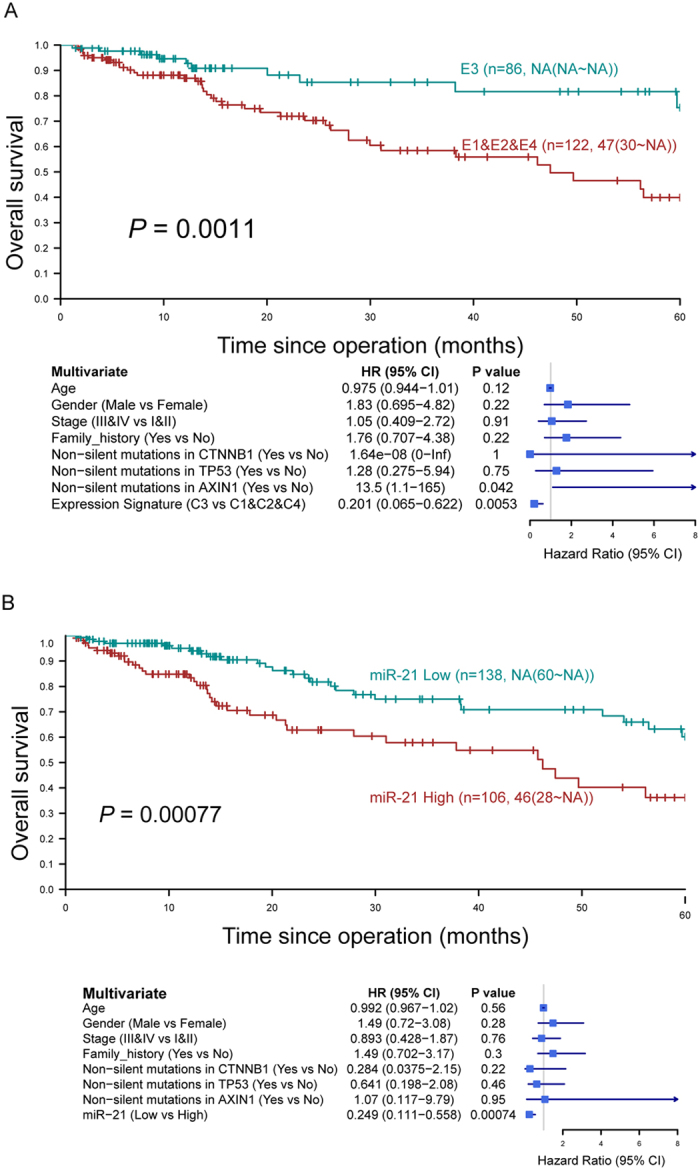
Expression signatures associated with survival outcomes in HCC. (**A**) Kaplan-Meier curves show the overall survival of HCC patients with E3 and non-E3 expression signatures. Patients with E3 expression signature exhibited a significantly better outcome compared to those with non-E3. Multivariable Cox regression analysis showed that the E3 expression signature was a prognostic factor independent of other clinicopathological and mutational parameters. (**B**) miR-21 was an independent prognostic marker for HCC and its low expression was associated with a better outcome.

To determine if miRNA expression pattern could be used for molecular typing, NMF-based unsupervised clustering was performed. Among 7 types of digestive cancer, specific subtypes of HCC and colon adenocarcinoma were identified to exhibit distinct survival characteristics. Patients treated with chemotherapy, radiation or transplantation were omitted from this analysis to avoid being confounded by treatment options. In HCC, 4 expression signatures (E1-4) could be identified, in which E3 exhibited a significantly better survival outcome as compared with non-E3 signatures (Fig. 5A; Supplementary Figure 6). Importantly, multivariable Cox regression analysis demonstrated that the expression signature could serve as a prognostic factor independent of age, gender and TNM staging as well as major somatic mutations (i.e. *CTNNB1*, *TP53*, *AXIN1*) identified in HCC. Random forest identified two miRNAs (i.e. miR-21 and miR-148a) with the greatest contribution to this molecular classification (Supplementary Figure 6) and both of them *per se* were associated with HCC patients’ survival but only miR-21 was an independent prognostic marker (Fig. 5B). To enhance the clinical utilization of our findings, a prediction formula was built to determine if a sample belongs to E3 based on the expression of miR-21 and miR-148a: ∆s = (−27.98) *log_2_(TPM of miR-21) + 32.51 *log_2_(TPM of miR-148a). The bigger the ∆s was, the more possible the sample belonged to E3 (AUC = 0.910; *p* < 0.01; Supplementary Figure 7). At a cutoff of 25.15, ∆s could classify a sample into E3/non-E3 with a sensitivity of 0.912 and a specificity of 0.771.

**Figure 6:**
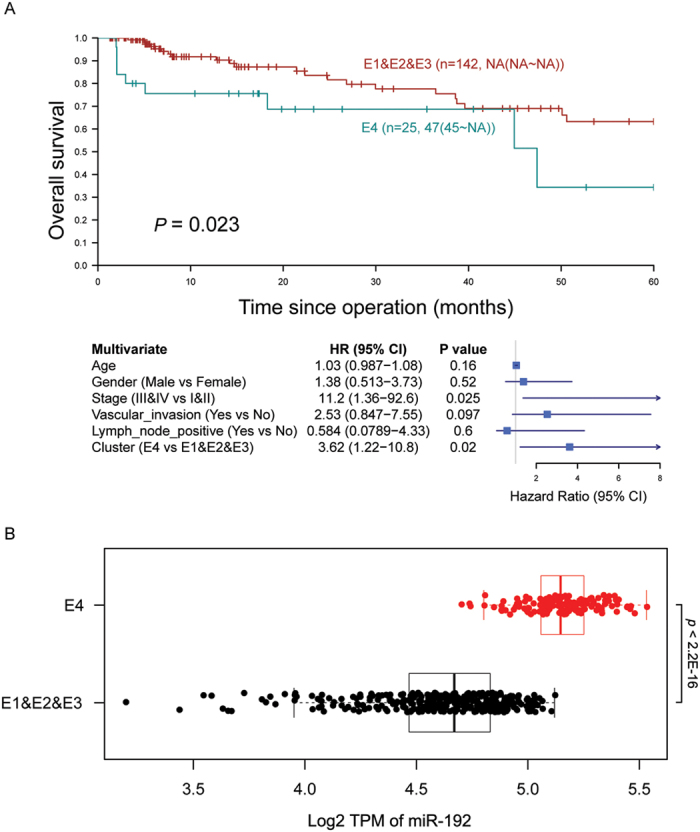
Expression signatures associated with survival outcomes in colon adenocarcinoma. (**A**) E4 expression signature was associated with a significantly worse survival outcome as compared with non-E4 signatures. (**B**) Differential expression of miR-192 could be detected between E4 and non-E4 tumors.

Similarly, in colon adenocarcinoma, 4 expression signatures (E1-4) could be identified (Supplementary Figure 8), in which E4 was found to be associated with a significantly worse survival outcome as compared with non-E4 signatures (Fig. 6A). Importantly, the prognostic significance of E4/non-E4 was independent of age, gender, vascular and lymph node invasion, and TNM staging (Fig. 6A) and the two groups could be distinguished by the expression level of miR-192 (Fig. 6B; Supplementary Figure 8). Nevertheless, since mutation profiles of most of the colon adenocarcinoma samples are unavailable, it would be difficult to assess if these markers are directly related to survival outcomes or merely downstream of some key genomic mutations.

### Specific miRNAs associated with survival

**Figure 7:**
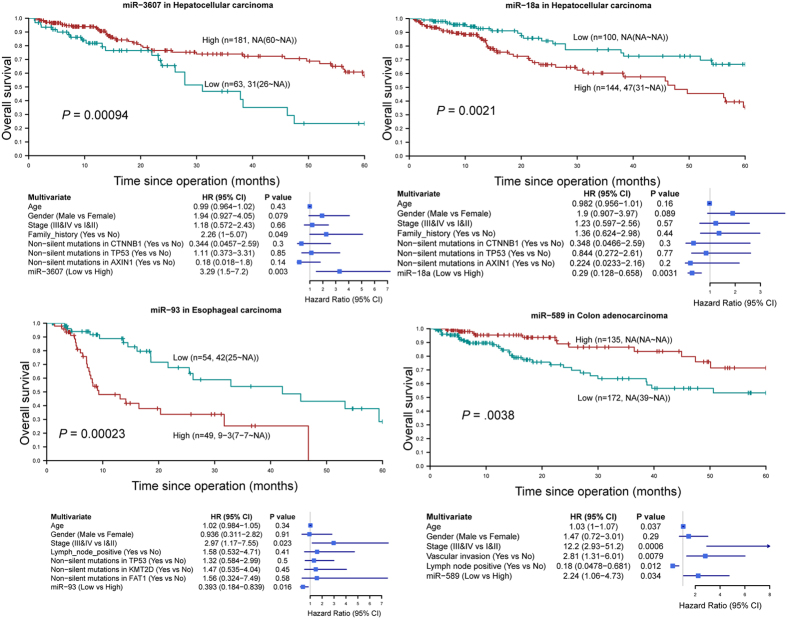
Four miRNAs as novel prognosticators independent of TNM staging and major somatic mutations in hepatocellular carcinoma, esophageal cancer and colon adenocarcinoma. Prognostic significance of each miRNA was assessed by Kaplan-Meier survival curve and the log-rank test on Youden index-derived high- and low-expression patient subgroups followed by univariate (not shown) and multivariate Cox regression analyses.

To identify miRNAs associated with patients’ overall survival independent of other clinicopathological parameters, we performed multivariate survival analysis for each miRNA. This approach identified 10 miRNAs as independent prognostic markers (Supplementary Table 1; Fig. 7; Supplementary Figure 9), among which high expression of 7 miRNAs foreshadowed poor prognosis: (1) miR-182, miR-18a, miR-21, miR-221, and miR-25 in HCC; (2) miR-215 in colon adenocarcinoma; (3) miR-93 in esophageal cancer; whereas low expression of 3 miRNAs predicted poor prognosis: (1) miR-3607 in HCC; (2) miR-140 and miR-589 in colon adenocarcinoma.

## Discussion

In this study, we analyzed 1765 tumor samples, including esophageal, gastric, liver, pancreatic, colon and rectal cancers, according to their miRNA expression profiles. Our results reveal that miRNAs can be used to distinguish their tissue origins, even colon from rectum. This is in line with a previous study that miRNA expression profiles could be used to predict tumor origin^19^. Notably, esophagus, gastric, liver and pancreatic cancers share higher similarities than colon and rectal cancers, consistent with the distinct midgut/hindgut origins of colon and rectum during embryonic development (Fig. 1A) and the reported role of miRNAs in terminal differentiation^20^,^21^,^22^. Moreover, we found that ESCC and EAC exhibit different miRNA expression patterns, in which the latter has a profile closely resembling that of gastric cancer, resonating with the finding that metaplasia in Barrett’s esophagus, a condition predisposing to EAC, is driven by the upward migration of stem cells from the proximal stomach^23^.

MSI is a hypermutable phenotype caused by deficiency in DNA mismatch repair and has been reported in colon, gastric, endometrial and ovarian cancers^24^,^25^,^26^,^27^. Herein, we demonstrate that MSI status is closely associated with miRNA expression patterns in colon and, to a lesser extent, gastric cancers. In both cancer types, miR-181d could be used to discriminate MSI-H from non-MSI-H samples. It is highly possible that low miR-181d expression in MSI-H samples is a compensatory response instead of the cause of MSI since miR-181d has been shown to target the DNA-repair enzyme O^6^-methylguanine-DNA methyltransferase (MGMT)^28^. In colon cancer, MSI tumors display chemosensitivity to irinotecan but chemoresistance to 5-fluorouracil-based therapy^29^,^30^. It would be interesting to determine if miR-181d could be used to predict responsiveness to these commonly used chemotherapeutics in colon cancer.

For prognostication, low let-7f-2 and high miR-106a expression were identified to correlate with disease progression in colon cancer. It is consistent with the finding that overexpression of LIN28B, which represses biogenesis of let-7 miRNAs, correlates with increased recurrence in colon cancer^31^. Likewise, low expression of FER1L4, which sponges and thereby reducing the availability of miR-106a, is predictive of poor prognosis in colon cancer^32^. Colon cancer with lymph node metastases also tends to have a higher expression of miR-106a than those without^33^. Nevertheless, a prospective clinical study is required in the future to validate the prognostic significance of these two miRNAs in colon cancer.

Molecular typing has changed the way by which oncologists quantify recurrence risk and predict survival in cancer patients. It has also influenced the criteria on which patients are selected for more aggressive chemotherapy^34^. Here we described subtypes of HCC and colon cancer that are linked to distinct patterns of miRNA expression and survival outcomes. Given the key role of miRNAs in tumorigenesis, tremendous effort has also been put forth to investigate the association between specific miRNAs and survival outcomes. In this study, we identified 10 miRNAs as independent prognostic markers in esophageal cancer, HCC and colon cancer. Among them, 6 miRNAs (miR-21, miR-25, miR-140, miR-182, miR-221, miR-215) have been reported to show concordant association with prognosis in the same cancer type^35^,^36^,^37^,^38^,^39^,^40^,^41^. Importantly, 4 miRNAs (miR-18a, miR-93, miR-589, miR-3607) whose association with patients’ survival has not been reported were identified. Importantly, the prognostic significance of these miRNAs was independent of somatic mutations commonly identified in respective cancer types (except colon cancer samples whose mutation profiles are unavailable). Thus these miRNAs could serve as novel prognostic biomarkers. However, it is noteworthy that samples collected from TCGA were usually biased towards early-stage, resectable tumors and therefore some late-stage markers may be missed by the current approach.

In summary, we conducted an integrative analysis of 1765 samples of digestive cancers based on their miRNA expression profiles, in which novel miRNAs for predicting tissue origins, pathological features and prognostic outcomes were identified. It is hopeful that some prognostic markers could help to identify patients with poorer predicted clinical outcomes for more aggressive treatment in the future.

## Materials and Methods

### miRNA expression data

All miRNA expression data and clinical information were collected from the TCGA open access data directory. (https://tcga-data.nci.nih.gov/tcgafiles/ftp_auth/distro_ftpusers/anonymous/tumor/). A total of 1,765 tumor samples and 123 normal samples with their miRNA expression profiles obtained by small RNA sequencing were included in the present study. Reads mapped to known miRNA stem-loops (level 3 data) were used to quantify miRNA expression level and normalized by transcripts per million mapped reads (TPM). Only miRNAs that were expressed in at least one digestive tissue/cancer were subject to further analyses. The criteria of defining a miRNA being expressed: (i) a miRNA was expressed at levels of at least 10 TPM in no less than 50% of the tumor samples or (ii) the average TPM in normal samples was no less than 10. For defining differentially expressed miRNAs, fold-change (tumors versus normal tissues) calculated by TPM or DESeq was used where appropriate^42^. Significantly differentially expressed miRNAs were those that had a fold-change >1.5 and an adjusted *p* value < 0.05 using Benjamini Hochberg method. The criterion of defining outlier miRNAs was based on the interquartile range. Given Q1 and Q3 are lower and upper quartiles, miRNAs with fold-change outside the range [Q1 − 1.5(Q3-Q1), Q3 + 1.5(Q3-Q1)] were defined as outlier miRNAs.

### Principal component analysis (PCA) and hierarchical clustering

PCA was performed using the log_2_-transformed values of fold-change to compare the miRNA expression profiles of different samples. The ade4 package in R program was used to perform PCA. For hierarchical clustering, only significantly differentially expressed miRNAs were included. Variable elimination from random forest was carried out to select miRNAs with the greatest contribution to the classification using the out-of-bag (OOB) error as a minimization criterion.

### Receiver-operating characteristic curve (ROC) and formulas for predicting clinical features

The area under the ROC curve (AUC) was used as a measure to evaluate the classification performance of selected miRNAs. To calculate AUC, tumor samples were divided into 2 equal subsets, namely the training group and the validation group. A predictable model using the expression levels of selected miRNAs of the training group was then built followed by putting those of the validation group into the model. The above steps were repeated 200 times (or 2000 times for constructed formulas) to calculate an average AUC. Mathematical formulas for predicting clinical features with miRNA expression data were constructed, which assigned each patient a score (∆s) according to a linear combination of log_2_(the expression level of the miRNAs), weighted by the regression coefficients (r): ∆s = ∑r *log_2_(the expression level of the miRNAs).

### miRNA typing

Unsupervised non-negative matrix factorization (NMF) was performed to classify samples into different subtypes based on miRNA profiles^43^. The Brunet algorithm and 100 iterations were used for the rank survey r from 2 to 10 clusters. The optimal value of r was chosen by the first value of r for which the cophenetic coefficient starts decreasing and the first value where the RSS curve presents an inflection point. Additionally, a visually clean consensus matrix was considered when the optimal value of r was selected.

### Survival analysis

Association of miRNA expression with patients’ overall survival was assessed by Kaplan-Meier survival curve and the log-rank test on Youden index-derived high- and low-expression patient subgroups for each miRNA. Univariate and multivariate Cox model were constructed to estimate hazard ratio for miRNAs with a *p* value less than 0.05 in the log-rank test.

## Additional Information

**How to cite this article**: Tang, S. *et al.* Stratification of Digestive Cancers with Different Pathological Features and Survival Outcomes by MicroRNA Expression. *Sci. Rep.* **6**, 24466; doi: 10.1038/srep24466 (2016).

## Supplementary Material

Supplementary Information

## References

[b1] Torre L. A., Bray F., Siegel R. L., Ferlay J., Lortet-Tieulent J. & Jemal A. Global cancer statistics. 2012. CA Cancer J. Clin. 65, 87–108 (2015). 25651787 10.3322/caac.21262

[b2] Wu W. K. & Sung J. J. Focus on gastrointestinal and liver cancers. Semin. Cancer Biol. 23, 469–470 (2013). 24095959 10.1016/j.semcancer.2013.09.006

[b3] Cancer Genome Atlas Research Network *et al.* Comprehensive molecular characterization of gastric adenocarcinoma. Nature 513, 202–209 (2014). 25079317 10.1038/nature13480 PMC4170219

[b4] Lin D. C. *et al.* Genomic and molecular characterization of esophageal squamous cell carcinoma. Nat. Genet. 46, 467–473 (2014). 24686850 10.1038/ng.2935 PMC4070589

[b5] Cancer Genome Atlas Network *et al.* Comprehensive molecular characterization of human colon and rectal cancer. Nature 487, 330–337 (2012). 22810696 10.1038/nature11252 PMC3401966

[b6] Wong S. H. *et al.* Genome-wide association and sequencing studies on colorectal cancer. Semin. Cancer Biol. 23, 502–511 (2013). 24096009 10.1016/j.semcancer.2013.09.005

[b7] Cristescu R. *et al.* Molecular analysis of gastric cancer identifies subtypes associated with distinct clinical outcomes. Nat. Med. 21, 449–456 (2015). 25894828 10.1038/nm.3850

[b8] Yu J. *et al.* Novel recurrently mutated genes and a prognostic mutation signature in colorectal cancer. Gut 64, 636–645 (2015). 24951259 10.1136/gutjnl-2013-306620 PMC4392212

[b9] Wu W. K. *et al.* MicroRNA dysregulation in gastric cancer: a new player enters the game. Oncogene 29, 5761–5771 (2010). 20802530 10.1038/onc.2010.352

[b10] Wu W. K. *et al.* MicroRNA in colorectal cancer: from benchtop to bedside. Carcinogenesis 32, 247–253 (2011). 21081475 10.1093/carcin/bgq243

[b11] Srivastava S. K., Arora S., Singh S., Bhardwaj A., Averett C. & Singh A. MicroRNAs in pancreatic malignancy: progress and promises. Cancer Lett. 347, 167–174 (2014). 24561061 10.1016/j.canlet.2014.02.015 PMC3989403

[b12] Callegari E., Gramantieri L., Domenicali M., D’Abundo L., Sabbioni S. & Negrini M. MicroRNAs in liver cancer: a model for investigating pathogenesis and novel therapeutic approaches. Cell Death Differ. 22, 46–57 (2015). 25190143 10.1038/cdd.2014.136 PMC4262781

[b13] Dong Y. *et al.* MicroRNA dysregulation in colorectal cancer: a clinical perspective. Br. J. Cancer 104, 893–898 (2011). 21364594 10.1038/bjc.2011.57 PMC3065287

[b14] Tang G., Shen X., Lv K., Wu Y., Bi J. & Shen Q. Different normalization strategies might cause inconsistent variation in circulating microRNAs in patients with hepatocellular carcinoma. Med. Sci. Monit. 21, 617–624 (2015). 25719241 10.12659/MSM.891028 PMC4345856

[b15] Shrestha S. *et al.* A systematic review of microRNA expression profiling studies in human gastric cancer. Cancer Med. 3, 878–888 (2014). 24902858 10.1002/cam4.246 PMC4303155

[b16] Gong X. *et al.* Evaluating the consistency of differential expression of microRNA detected in human cancers. Mol. Cancer Ther. 10, 752–60 (2011). 21398424 10.1158/1535-7163.MCT-10-0837

[b17] Williams Z. *et al.* Comprehensive profiling of circulating microRNA via small RNA sequencing of cDNA libraries reveals biomarker potential and limitations. Proc. Natl. Acad. Sci. USA 110, 4255–4260 (2013). 23440203 10.1073/pnas.1214046110 PMC3600502

[b18] Law P. T. *et al.* Deep sequencing of small RNA transcriptome reveals novel non-coding RNAs in hepatocellular carcinoma. J. Hepatol. 58, 1165–1173 (2013). 23376363 10.1016/j.jhep.2013.01.032

[b19] Rosenfeld N. *et al.* MicroRNAs accurately identify cancer tissue origin. Nat. Biotechnol. 26, 462–469 (2008). 18362881 10.1038/nbt1392

[b20] Pobezinsky L. A. *et al.* Let-7 microRNAs target the lineage-specific transcription factor PLZF to regulate terminal NKT cell differentiation and effector function. Nat. Immunol. 16, 517–524 (2015). 25848867 10.1038/ni.3146 PMC4406853

[b21] de la Rica L. *et al.* NF-kappaB-direct activation of microRNAs with repressive effects on monocyte-specific genes is critical for osteoclast differentiation. Genome Biol. 16, 2 (2015). 25601191 10.1186/s13059-014-0561-5 PMC4290566

[b22] Lu D. *et al.* The miR-155-PU.1 axis acts on Pax5 to enable efficient terminal B cell differentiation. J. Exp. Med. 211, 2183–2198 (2014). 25288398 10.1084/jem.20140338 PMC4203942

[b23] Quante M. *et al.* Bile acid and inflammation activate gastric cardia stem cells in a mouse model of Barrett-like metaplasia. Cancer Cell 21, 36–51 (2012). 22264787 10.1016/j.ccr.2011.12.004 PMC3266546

[b24] Kim T. M., Laird P. W. & Park P. J. The landscape of microsatellite instability in colorectal and endometrial cancer genomes. Cell 155, 858–868 (2013). 24209623 10.1016/j.cell.2013.10.015 PMC3871995

[b25] Popat S., Hubner R. & Houlston R. S. Systematic review of microsatellite instability and colorectal cancer prognosis. J. Clin. Oncol. 23, 609–618 (2005). 15659508 10.1200/JCO.2005.01.086

[b26] Singer G. *et al.* Different types of microsatellite instability in ovarian carcinoma. Int. J. Cancer 112, 643–646 (2004). 15382045 10.1002/ijc.20455

[b27] Halling K. C. *et al.* Origin of microsatellite instability in gastric cancer. Am. J. Pathol. 155, 205–211 (1999). 10393852 10.1016/S0002-9440(10)65114-0 PMC1866662

[b28] Zhang W. *et al.* miR-181d: a predictive glioblastoma biomarker that downregulates MGMT expression. Neuro. Oncol. 14, 712–719 (2012). 22570426 10.1093/neuonc/nos089 PMC3367855

[b29] Jo W. S. & Carethers J. M. Chemotherapeutic implications in microsatellite unstable colorectal cancer. Cancer Biomark. 2, 51–60 (2006). 17192059 10.3233/cbm-2006-21-206 PMC4948976

[b30] Fallik D. *et al.* Microsatellite instability is a predictive factor of the tumor response to irinotecan in patients with advanced colorectal cancer. Cancer Res. 63, 5738–5744 (2003). 14522894

[b31] King C. E., Cuatrecasas M., Castells A., Sepulveda A. R., Lee J. S. & Rustgi A. K. LIN28B promotes colon cancer progression and metastasis. Cancer Res. 71, 4260–4268 (2011). 21512136 10.1158/0008-5472.CAN-10-4637 PMC3117110

[b32] Yue B. *et al.* Long non-coding RNA FER1L4 suppresses oncogenesis and exhibits prognostic value by associating with miR-106a-5p in colon cancer. Cancer Sci. 106, 1323–1332 (2015). 26224446 10.1111/cas.12759 PMC4638023

[b33] Ak S. *et al.* MicroRNA expression patterns of tumors in early-onset colorectal cancer patients. J. Surg. Res. 191, 113–122 (2014). 24746948 10.1016/j.jss.2014.03.057

[b34] Kittaneh M., Montero A. J. & Gluck S. Molecular profiling for breast cancer: a comprehensive review. Biomark. Cancer 5, 61–70 (2013). 10.4137/BIC.S9455 PMC3825646 24250234

[b35] Zhai H., Fesler A., Ba Y., Wu S. & Ju J. Inhibition of colorectal cancer stem cell survival and invasive potential by hsa-miR-140-5p mediated suppression of Smad2 and autophagy. Oncotarget 6, 19735–19746 (2015). 25980495 10.18632/oncotarget.3771 PMC4637317

[b36] Cai L. & Cai X. Up-regulation of miR-9 expression predicate advanced clinicopathological features and poor prognosis in patients with hepatocellular carcinoma. Diagn. Pathol. 9, 1000 (2014). 25552204 10.1186/s13000-014-0228-2 PMC4348155

[b37] Wang W. Y. *et al.* miR-21 expression predicts prognosis in hepatocellular carcinoma. Clin. Res. Hepatol. Gastroenterol. 38, 715–719 (2014). 25150373 10.1016/j.clinre.2014.07.001

[b38] Su Z. X., Zhao J., Rong Z. H., Geng W. M., Wu Y. G. & Qin Q. K. Upregulation of microRNA-25 associates with prognosis in hepatocellular carcinoma. Diagn. Pathol. 9, 47 (2014). 24593846 10.1186/1746-1596-9-47 PMC4016611

[b39] Wang J., Li J., Shen J., Wang C., Yang L. & Zhang X. MicroRNA-182 downregulates metastasis suppressor 1 and contributes to metastasis of hepatocellular carcinoma. BMC Cancer 12, 227 (2012). 22681717 10.1186/1471-2407-12-227 PMC3492170

[b40] Karaayvaz M. *et al.* Prognostic significance of miR-215 in colon cancer. Clin. Colorectal Cancer 10, 340–347 (2011). 21752725 10.1016/j.clcc.2011.06.002 PMC3390153

[b41] Li J., Wang Y., Yu W., Chen J. & Luo L. Expression of serum miR-221 in human hepatocellular carcinoma and its prognostic significance. Biochem. Biophys. Res. Commun. 406, 70–73 (2011). 21295551 10.1016/j.bbrc.2011.01.111

[b42] Anders S. & Huber W. Differential expression analysis for sequence count data. Genome Biol. 11, R106 (2010). 20979621 10.1186/gb-2010-11-10-r106 PMC3218662

[b43] Gao Y. & Church G. Improving molecular cancer class discovery through sparse non-negative matrix factorization. Bioinformatics 21, 3970–3975 (2005). 16244221 10.1093/bioinformatics/bti653

