# Supplementary material for: Stratification of Digestive Cancers with Different Pathological Features and Survival Outcomes by MicroRNA Expression
Source: Sci Rep. 2016 Apr 15;6:24466. doi: 10.1038/srep24466 (PMC4832245; doi:10.1038/srep24466)
Supplement: Supplementary Information [file srep24466-s1.pdf]

Supplementary Information

Manuscript Title: Stratification of Digestive Cancers with Different Pathological Features and Survival Outcomes by MicroRNA Expression

Authors: Senwei Tang, William K.K. Wu, Xiangchun Li, Sunny H. Wong, Nathalie Wong, Matthew T.V. Chan, Joseph J.Y. Sung, Jun Yu

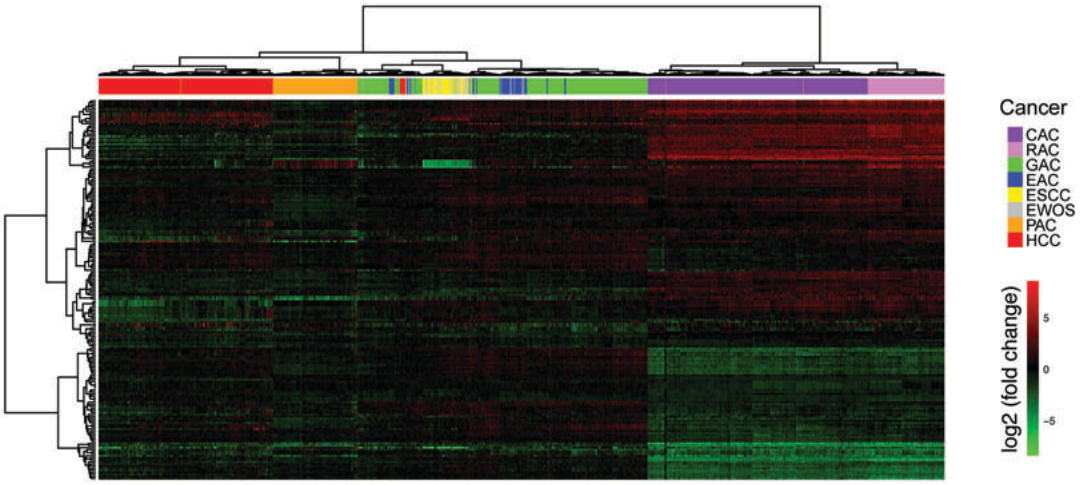

Supplementary Figure 1 Hierarchical clustering of 1765 tumor samples using 378 miRNAs expressed in at least one type of digestive tissue/cancer. CAC, colon adenocarcinoma; RAC, rectal adenocarcinoma; GAC, gastric adenocarcinoma; EAC, esophageal adenocarcinoma; ESCC, esophageal squamous cell carcinoma; EWOS, esophageal cancer not otherwise specified; PAC, pancreatic adenocarcinoma; HCC, hepatocellular carcinoma.

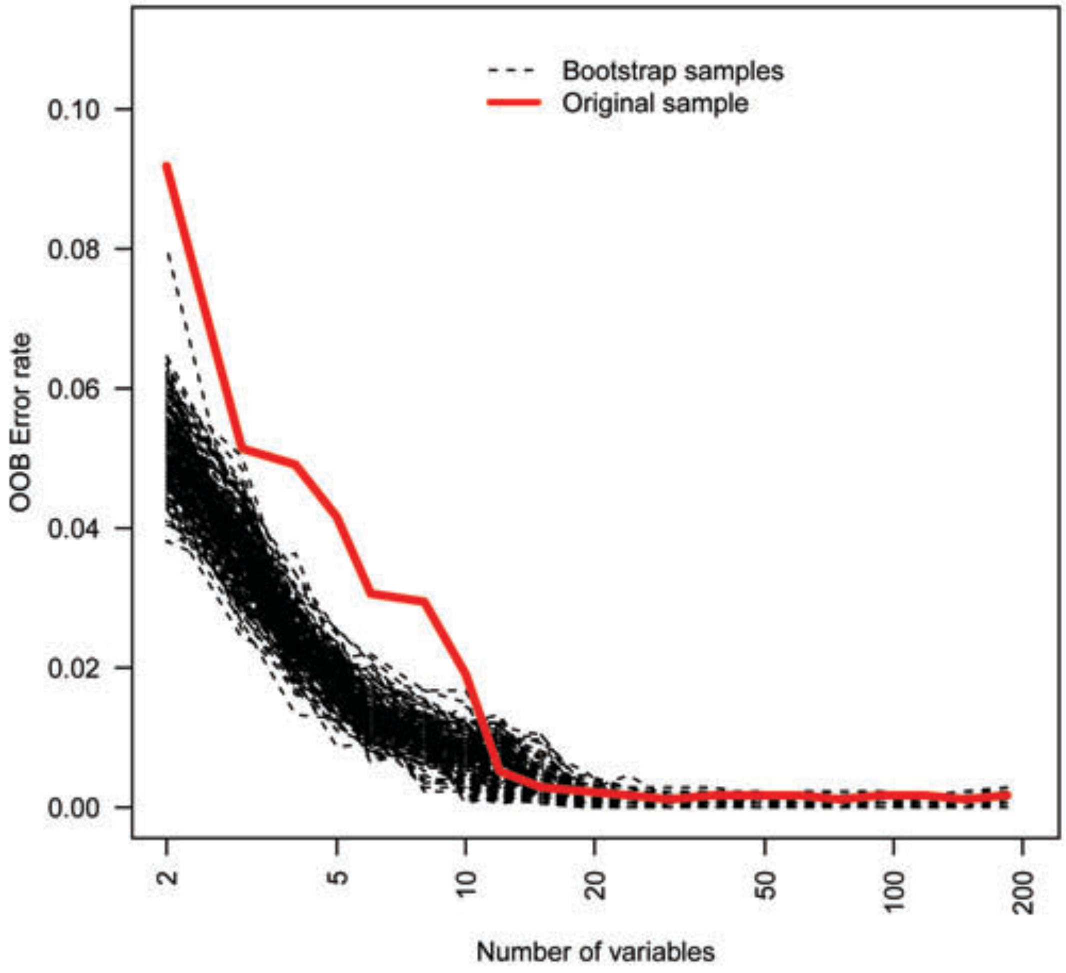

Supplementary Figure 2 Out-of-Bag (OOB) error rate versus number of variables in predictor. OOB error rate was minimal when the number of variables equaled 34.

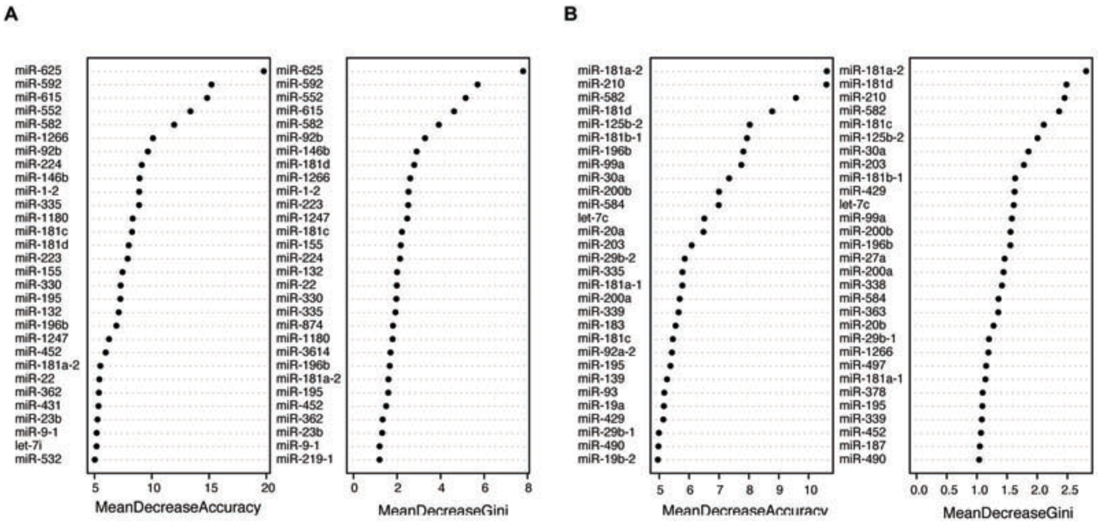

Supplementary Figure 3 Random Forest for selection of miRNAs with greatest contribution to MSI classification in (**a**) colon adenocarcinoma and (**b**) gastric adenocarcinoma.

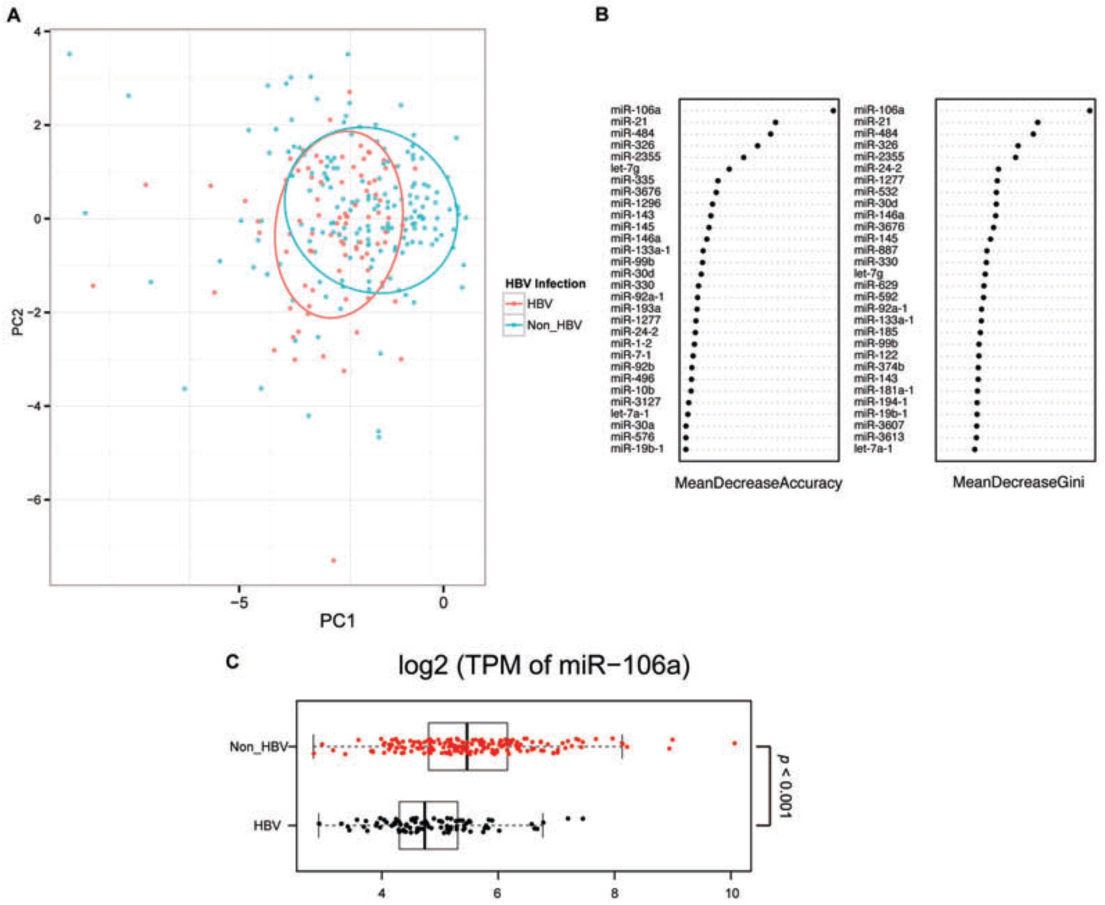

Supplementary Figure 4 Correlation between miRNA expression and HBV status in HCC. (**a**) Principal component analysis shows the miRNA expression profiles in HBV versus non-HBV HCC samples. (**b**) Random forest was used to select miRNAs with greatest contribution to HBV status classification. (**c**) miR-106a expression levels were significantly lower in HBV HCC samples as compared with non-HBV HCC samples.

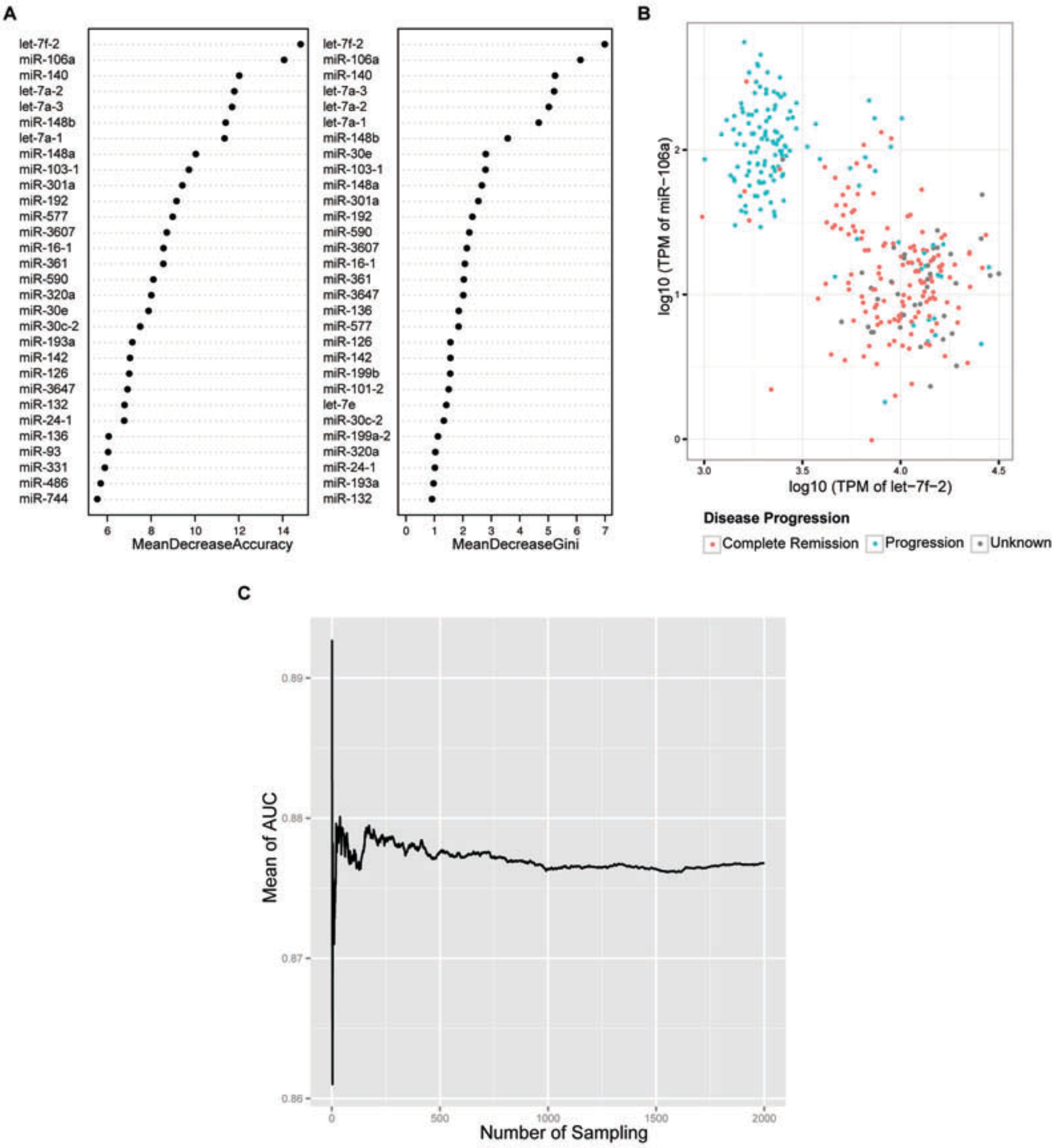

Supplementary Figure 5 Random forest-selected miRNAs for distinguishing colon adenocarcinomas with complete remission from those with disease progression. (**a**) The relative importance of miRNAs in disease status classification. (**b**) Expression levels of miR-106a and let-7f-2 in relation to disease status in colon adenocarcinoma. (**c**) Relationship between average AUC and the number of sampling.

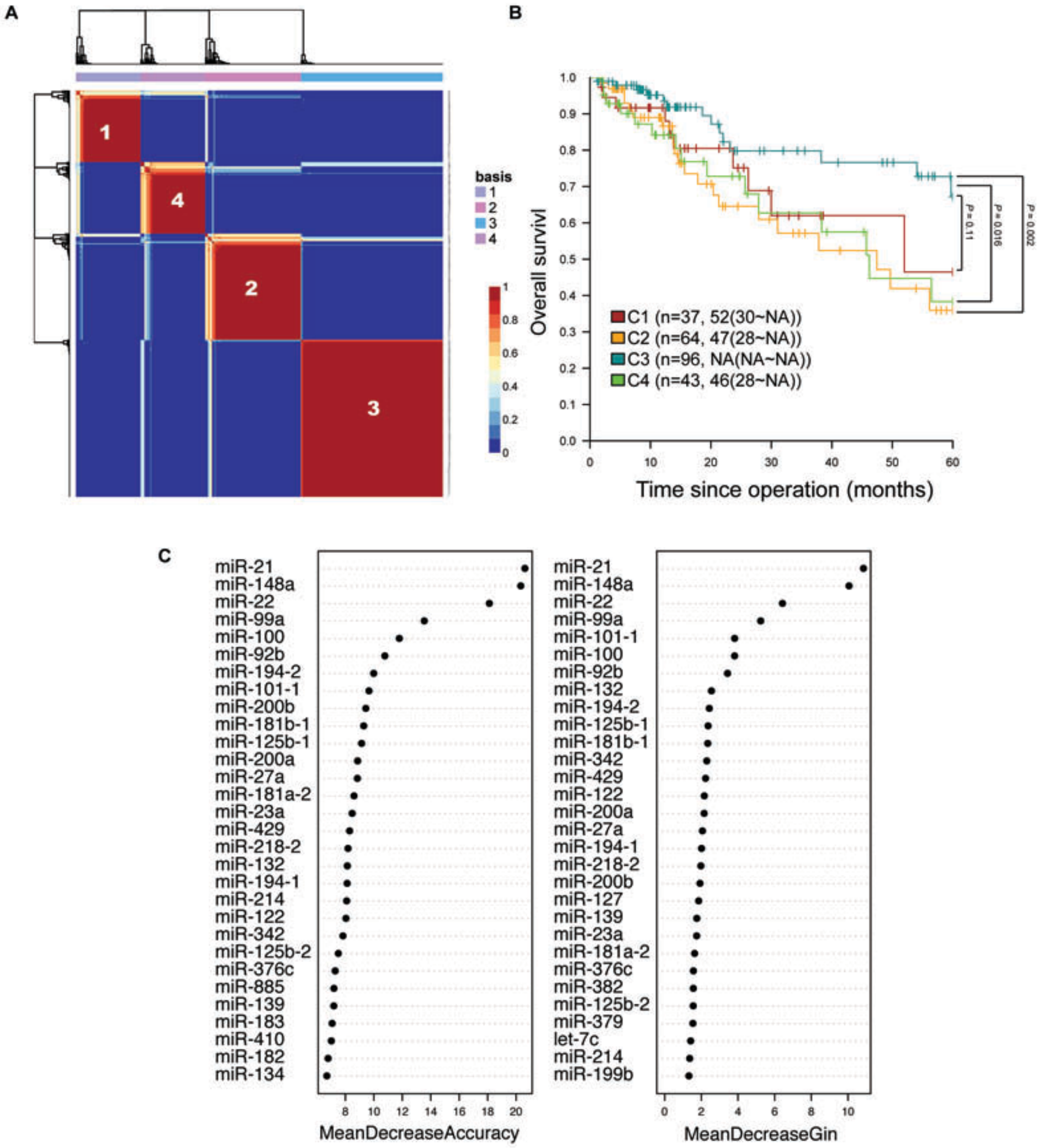

Supplementary Figure 6 miRNA expression signatures in HCC patients associated with distinct prognostic outcomes. (**a**) Identification of four miRNA expression signatures by nonnegative matrix factorization (NMF) clustering in HCC patients. (**b**) Kaplan-Meier curves showing overall survival in HCC patients with different miRNA expression signatures. (**c**) Identification of miRNAs with greatest contribution to E3/non-E3 classification by random forest.

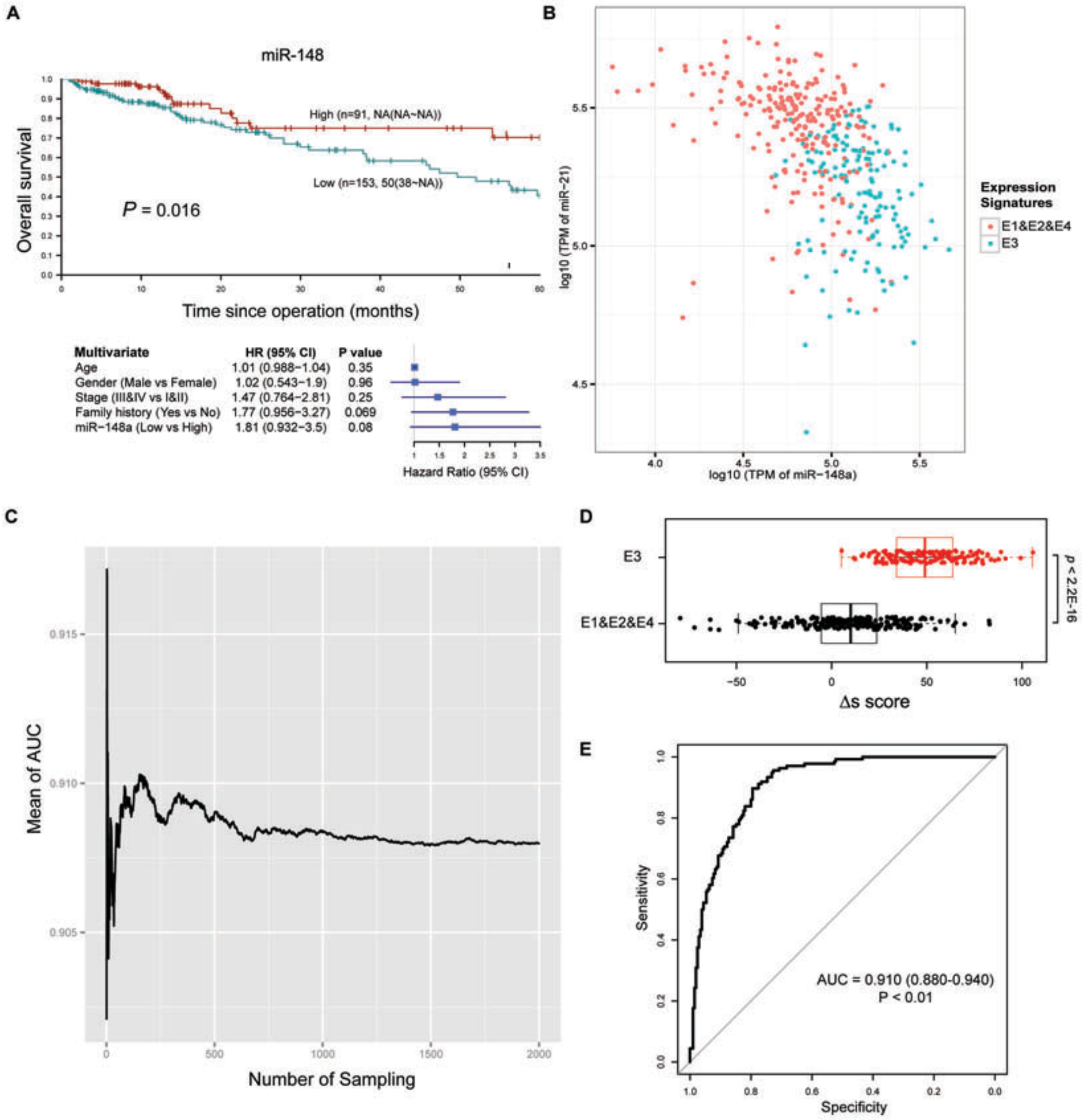

Supplementary Figure 7 Comparison between E3 with non-E3 expression signatures in HCC. (**a**) miR-148a was associated with HCC patients' survival but was not an independent prognostic marker. (**b**) Tumors with E3 expression signature had different expression profiles of miR-21 and miR-148a compared with non-E3 samples. (**c**) Relationship between average AUC and the number of sampling. (**d**) Δs score was significantly higher in E3 samples. (**e**) ROC curve was used to evaluate the performance of Δs score as a classifier of E3/non-E3 HCC samples.

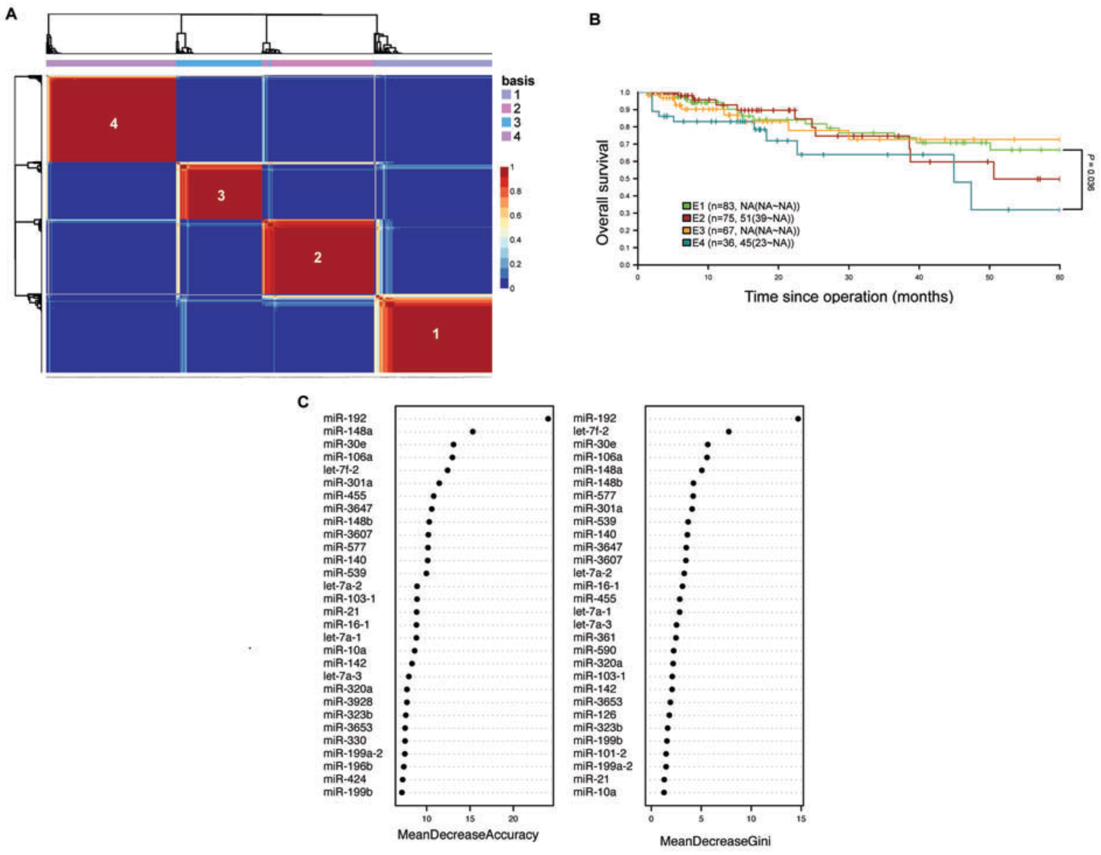

Supplementary Figure 8 Subtyping in colon adenocarcinoma based on NMF clustering of miRNA expression profiles. (**a**) NMF identified four expression signatures in patients with colon adenocarcinoma. (**b**) Kaplan-Meier curves showing overall survival in colon adenocarcinoma patients with different miRNA expression signatures. (**c**) Identification of miRNAs with greatest contribution to E4/non-E4 classification by random forest.

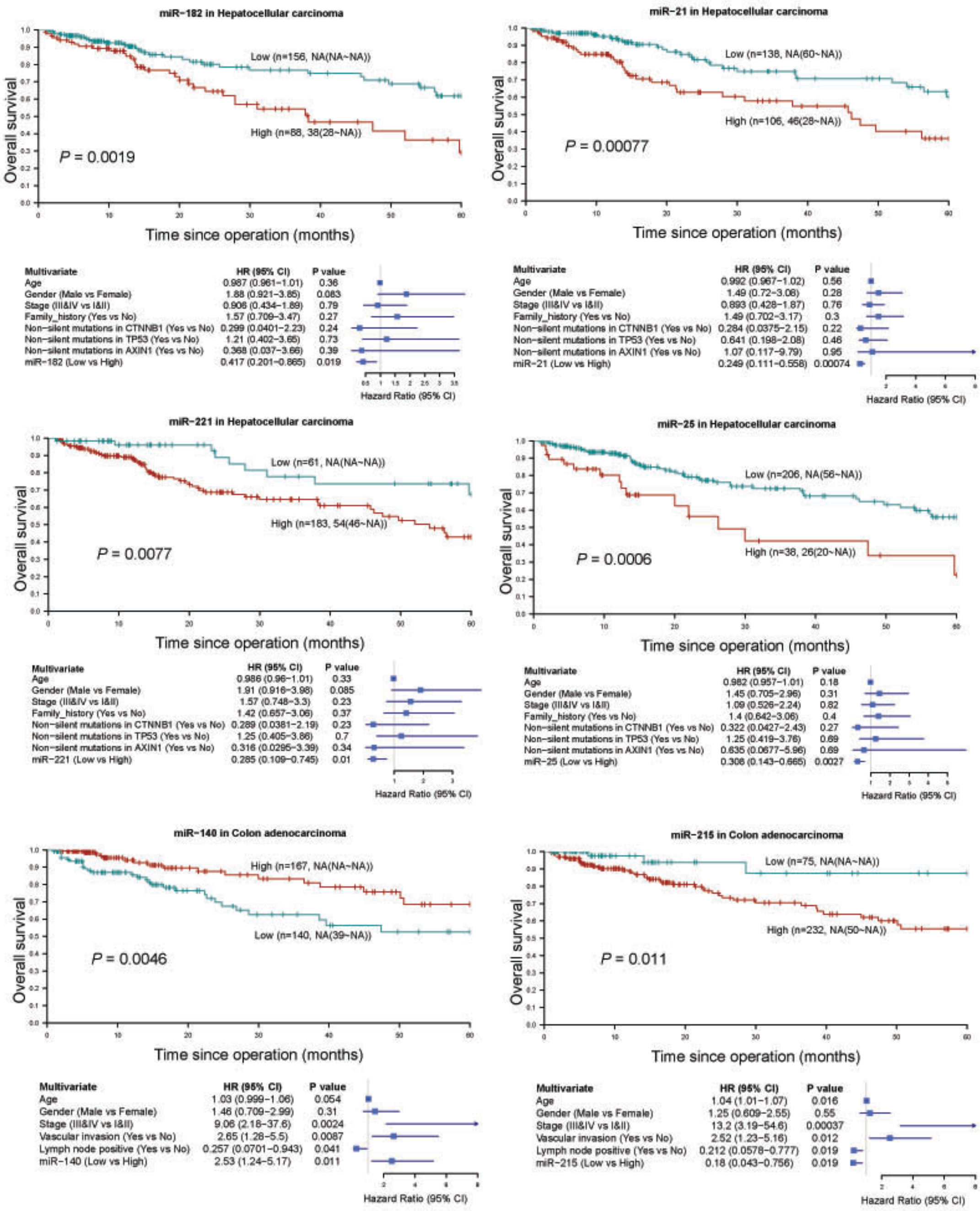

Supplementary Figure 9 Re-discovery of six independent prognostic miRNAs in hepatocellular carcinoma and colon adenocarcinoma. Prognostic significance of each miRNA was assessed by Kaplan-Meier survival curve and the log-rank test on Youden index-derived high- and low-expression patient subgroups followed by univariate (not shown) and multivariate Cox regression analyses.

Supplementary Table 1. Identification of 10 miRNAs as independent prognostic markers in hepatocellular carcinoma (HCC), esophageal cancer (EC), and colon adenocarcinoma (CAC).

**miRNA**	**Cancer**	**Poor prognosis**
miR-3607	HCC	Low
miR-182	HCC	High
miR-18a	HCC	High
miR-21	HCC	High
miR-221	HCC	High
miR-25	HCC	High
miR-93	EC	High
miR-140	CAC	Low
miR-589	CAC	Low
miR-215	CAC	High
